# Pro-coagulant activity during exercise testing in patients with coronary artery disease

**DOI:** 10.1186/s12959-016-0127-8

**Published:** 2017-01-19

**Authors:** Joanna Cwikiel, Ingebjorg Seljeflot, Eivind Berge, Harald Arnesen, Kristian Wachtell, Hilde Ulsaker, Arnljot Flaa

**Affiliations:** 1 Department of Cardiology, Center for Clinical Heart Research, Oslo University Hospital Ullevaal, PB 4956 Nydalen, 0424 Oslo, Norway; 2 Department of Cardiology, Oslo University Hospital Ullevaal, Oslo, Norway; 3 Faculty of Medicine, University of Oslo, Oslo, Norway; 4 Section of Cardiovascular and Renal research, Oslo University Hospital Ulleval, Oslo, Norway; 5 Department of Cardiology, Division of Cardiovascular and Pulmonary diseases, Oslo University Hospital, Oslo, Norway; 6 Modum Bad, Vikersund, Norway

**Keywords:** Coagulation, Atherosclerosis, Angina, Exercise testing, Coronary angiography

## Abstract

**Background:**

Strenuous exercise may trigger myocardial infarction through increased pro-coagulant activity. We aimed to investigate whether patients referred for exercise testing, who were found to have angiographically verified coronary artery disease (CAD), have a more hypercoagulable profile during exercise testing than those without CAD.

**Methods:**

Patients with symptoms of stable CAD were examined with exercise electrocardiography on bicycle ergometer. Venous blood samples were taken at rest and within 5 min after end of exercise. The following haemostatic variables were analyzed: tissue factor pathway inhibitor (TFPI) activity and antigen, prothrombin fragment 1 + 2 (F1 + 2), D-dimer and endogenous thrombin potential (ETP). All participants underwent conventional coronary angiography. CAD was defined as having any degree of atherosclerosis.

**Results:**

Out of the 106 patients enrolled, 70 were found to have CAD. Mean exercise duration was 10:06 ± 4:11 min, with no significant differences between the groups. A significant increase from baseline to after exercise testing was observed in all measured markers in the total population (*p* ≤ 0.002 for all). In patients with angiographically verified CAD, total TFPI was significantly lower at baseline compared to patients without CAD (median value 67.4 and 76.6 ng/ml respectively, *p* = 0.027). However, no significant differences in changes of any of the measured markers during exercise were observed between the two groups.

**Conclusion:**

Pro-coagulant activity increased during short-term strenuous exercise testing in patients with symptoms suggestive of CAD. However the hypercoagulable state observed, was not more pronounced in patients with angiographically verified CAD compared to patients without CAD. NCT01495091.

## Background

Atherosclerotic coronary artery disease (CAD) is a chronic inflammatory process caused by accumulation of low-density lipoproteins (LDL) and plaque formation, activation of intimal inflammation and immune response initiated by endothelial injury and dysfunction, and activation of the haemostatic system [1]. These vascular alterations with subsequent plaque instability may lead to an acute coronary event with fatal consequences. While coronary artery plaques are known to develop over several years, the haemostatic activation is thought to be more prominent in the acute phase of a myocardial infarction [2].

With endothelial injury at plaque site, the haemostatic process is initiated with platelet activation by collagen and von Willebrand factor in the vessel wall, and simultaneously, tissue factor (TF) from the necrotic core of the plaque binds to factor VII, inducing the coagulation cascade [3, 4]. Tissue factor pathway inhibitor (TFPI) synthesized by vascular endothelial, smooth muscle cells and possibly by platelets [5], is the main inhibitor of TF-mediated coagulation, thus mainly reflecting anticoagulant activity. Prothrombin fragment 1 + 2 (F1 + 2) which is generated through the conversion of prothrombin to thrombin reflects the amount of in vivo thrombin formed, while on-going coagulation and fibrinolysis can be assessed by D-dimer, a fibrin degradation product. Ex vivo thrombin generation potential, which lately has been given attention as a measure of the degree of hypercoagulability, can be estimated through endogenous thrombin potential (ETP). The ETP is provided through a thrombogram measuring a set of parameters reflecting speed and amount of thrombin generated after standardized activation [6].

It is well recognized that strenuous physical exercise may provoke symptoms of angina or an acute coronary syndrome. During acute heavy physical load simultaneous activation of the coagulation and fibrinolytic processes is believed to occur. An exercise induced transient hypercoagulable state is known to take place in all individuals [7, 8], especially in those who are untrained [9]. Previous studies have suggested an imbalance between coagulation and fibrinolysis in favor of coagulation, in patients with CAD during strenuous exercise [10]. Changes in various haemostatic markers during heavy physical load have previously been investigated, mainly focusing on F1 + 2 and D-dimer [9, 11–13], although mapping of these is far from complete. Previous studies with observations of increased amounts of free and total TFPI in CAD have mainly focused on patients with acute coronary syndrome while sparse studies include patients with stable angina [14–16].

To provide more insight into mechanisms of haemostatic activity among patients with CAD, the aim of our study was to investigate whether patients with angiographically verified CAD, undergoing strenuous exercise, have an increase in markers of pro-coagulant activity different from those without verified CAD.

## Methods

### Study population

Patients referred for exercise testing due to symptoms suspected of stable CAD enrolled in the on-going CADENCE study (clinicaltrials.gov NCT01495091) at the outpatient clinic at the Department of Cardiology, Oslo University Hospital Ullevaal, Oslo Norway. Eligible patients were those ≥ 18 years of age, with symptoms suspected of CAD and intermediate or high risk (Morise risk score [17] ≥ 9 points). Exclusion criteria were the following: acute coronary syndrome, clinical heart failure, on-going arrhythmia or implanted pacemaker, moderate to severe valvular heart disease, renal insufficiency (S-creatinine >150 μmol/L), inability to perform exercise testing or coronary angiography. For the purpose of the present investigation, patients on oral anticoagulant therapy were excluded. All participants have given written informed consent to participate. The study has been conducted in accordance with the Declaration of Helsinki, and the Regional Ethics Committee in South Eastern Health Region in Norway approved the protocol.

A thorough medical history was recorded before inclusion. Prior to exercise testing a physical examination including blood pressure, weight and waist circumference was performed. Hypertension and hyperlipidemia were defined according to known diagnosis or use of specific medication.

### Exercise stress test

Exercise testing was performed using an electrical bicycle ergometer, monitored by physician and nursing staff. Registration of a resting 12-lead ECG was taken before exercise, while continuous 12-lead ECG monitoring using a computerized electrocardiogram was used during the test. According to protocol the initial workload was 30 watts (W) for women and 50 W for men, with a gradual increase of 10 W per min and participant maintaining a pedaling rate (cadence) of about 65 rpm. Every third min patients were asked about their perceived exhaustion using Borg scale [18]. Auscultatory blood pressure measurements were performed every third min of the test. One physician assessed the test results. Patients were exercised to exhaustion, if there were no clinical signs of ischemia developed prior to reaching high intensity level. The test was stopped after a recovery time of 5 min. A positive test result was defined as having horizontal or down-sloping ST-segment > 1.0 mm (0.1 mV) at 60 milliseconds after the J point and/or chest pain or discomfort. Reasons for terminating the test were development of suspected pathological ECG changes such as ST-segment elevation, ST-segment depression in leads without Q waves, arrhythmias increasing through exercise, chest pain, desire of patient to stop the test and insufficient chronotropic response to exercise, insufficient or exaggerative hypertensive response (systolic blood pressure ≥ 250 mmHg or diastolic blood pressure ≥ 115 mmHg) [19].

### Blood sampling and laboratory methods

Blood samples were collected prior to exercise at rest and within 5 min of terminating workload, while patients were still seated on the bicycle ergometer. Citrated blood (0.129 M trisodium citrate in dilution 1:10) was separated within 30 min by centrifugation at 2500 x g at 4 °C and kept frozen at ÷80 °C until analyzed. The following commercially available enzyme immunoassays were used to determine levels of TFPI, F1 + 2 and D-dimer: Asserachrom free and total TFPI antigen, recognizing full-length and truncated TFPI molecules associated to lipoproteins, respectively (Stago Diagnostica, Asniere, France), Enzygnost® F1 + 2 (monoclonal) (Siemens, Marburg, Germany) and Asserachrom D-dimer (Stago Diagnostica). The inter assay coefficients of variation (CV) in our laboratory were 5.6, 5.7, 4.9 and 6.7%, respectively. ETP was determined by the Calibrated Automated Thrombogram (CAT) assay according to the manufacturer’s instructions (Thrombinoscope BV, Maastricht, The Netherlands) and thrombin generation was analyzed on the Fluoroscan Ascent fluorometer (Thermo Fisher Scientific OY, Vantaa, Finland). A reagent mixture of rTF and phospholipids in addition to a thrombin-specific fluorogenic substrate in Hepes buffer containing CaCl_2_ was added to the plasma to obtain a final concentration of 5 pM, 4 μM and 2.5 mM, respectively. To calculate the final results, plasma was measured together with a thrombin calibrator. The software (version 3.0.0.29; Thrombinoscope BV) enabled the calculation of the lag time (LT), peak thrombin generation (pTG), ETP (area under the curve) and time to peak (TTP). Further, Velo (Velocity Index) = TP/(TTP-LT), indicating the average net rate of prothrombin activation during the propagation phase. All experiments were run in duplicates. The inter assay CVs for the different CAT variables were 14.2, 4.6, 5.0 and 8.0%, respectively.

### Coronary angiography

All study participants were referred to coronary angiography, performed using the standard Seldinger technique, mostly by using radial artery access. Quantitative coronary angiography (QCA) was analyzed in all angiograms by a single investigator. According to protocol, if a luminal artery stenosis was ≥30%, fractional flow reserve (FFR) was to be performed. Significant coronary stenosis was considered when minimal luminal diameter was >50% according to QCA measures or FFR ≤0.80.

For the purpose of this study CAD was defined as any degree of angiografically verified atherosclerosis, i.e. coronary angiographies described by the operator as having either minimal atherosclerotic changes to significant stenosis.

### Statistical analysis

Data was analyzed using IBM SPSS Statistics version 22.0. Laboratory values were mainly not normally distributed and are therefore presented with median value, 25th and 75th percentiles. Continuous data are otherwise presented as mean and standard deviation, and categorical data are presented as numbers (%). Depending on distribution of the continuous data either Student T-test or Mann–Whitney U test were used for comparisons between groups, while Wilcoxon signed rank test was used for pairwise comparisons of continuous data within the groups. For assessment of categorical variables Chi-square test was used. *P*-values ≤ 0.05 were considered statistically significant.

## Results

### Demographic and angiography data

Demographic, clinical and medical characteristics are described in Table 1, in the total cohort and according to having CAD or not. In total 120 of the patients enrolled in the CADENCE study were considered for this study of whom 3 patients were excluded due to anticoagulant therapy, 6 patients resigned before completing the study protocol, 5 patients were found to have ≥ 1 exclusion criteria not seen prior to inclusion. Remaining patients (*n* = 106) constituted the effective study sample. Out of the total population, 70 (66%) patients were found to have angiographically verified CAD and 28 (26.4%) patients were revascularized with either percutaneous coronary intervention or coronary artery bypass graft. The majority of the patients had one or more cardiovascular risk factors such as smoking (58%), hypertension (63%) and hyperlipidemia (79%), which were not significantly differently distributed between the two groups. A significantly lower number of men were found in the group without CAD compared to those with CAD (*p* = 0.012). Previous history of CAD was observed significantly more common in patients also diagnosed with CAD in this study. This is also illustrated by a significantly larger number of patients with CAD being treated with aspirin, statins or beta blockers (*p* ≤ 0.03 for all) compared to those without CAD (Table 1).

**Table 1:** Baseline characteristics of the total population and stratified into groups according to angiographically verified CAD or not

	Overall (*n* = 106) (%)	CAD (*n* = 70)	No CAD (*n* = 36)	*P*-value
Age (years)	62 ± 10	63 ± 10	60 ± 10	0.132
Sex (male)	62 (58.5)	47 (67.1)	15 (41.7)	*0.012*
Smoking (current/past)(%)	61 (57.5)	40 (57.1)	21 (58.3)	0.907
Diabetes (%)	19 (17.9)	15 (21.4)	5 (11.1)	0.190
Hypertension (%)	67 (63.2)	44 (62.9)	23 (63.9)	0.917
Hyperlipidemia (%)	84 (79.2)	59 (84.3)	25 (69.4)	0.074
BMI (kg/m^2^)	27.8 ± 4	27.9 ± 3.9	27.6 ± 3.8	0.726
Previous CAD (%)				
1.Previous angina	31 (29.2)	27 (38.6)	4 (11.1)	*0.003*
2.Previous MI	14 (13.2)	12 (17.1)	2 (5.6)	0.132
3.Previous interv.	27 (25.5)	25 (35.7)	2 (5.6)	*0.001*
Resting SBP (mmHg)	133 ± 22	133 ± 22	133 ± 21	0.928
Resting DBP (mmHg)	84 ± 10	84 ± 11	85 ± 9	0.621
Resting heart rate (bpm)	70 ± 13	68 ± 12	73 ± 13	0.050
ACE-inhib./ARB	43 (40.6)	30 (42.9)	13 (36.1)	0.503
Betablocker	45 (42.5)	35 (50)	10 (27.8)	*0.028*
Nitrate	9 (8.5)	9 (12.9)	0	*0.025*
Statin	68 (64.2)	50 (71.4)	18 (50)	*0.029*
Aspirin	71 (67)	55 (78.6)	16 (44.4)	*<0.001*

### Exercise performance

Mean exercise duration was 10:06 ± 4:11 min, exercise capacity 134 ± 48 W and mean metabolic equivalent (MET) 6.7 ± 1.8, with non-significant differences between the two groups. Positive exercise test results were found in 31 (44.3%) patients with CAD and in 7 (19.4%) patients without CAD. Comparable results were found between the revascularized (*n* = 28) versus non-revascularized (*n* = 78) groups performing an exercise test, with a sensitivity of 39.3% and specificity of 73.1% for CAD. Maximal heart rate was significantly lower in patients with CAD (137 ± 21 beats per minute (bpm)), Compared to those without CAD (150 ± 19 bpm) (*p* = 0.003).

### Markers of pro-coagulant activity

**Fig. 1:**
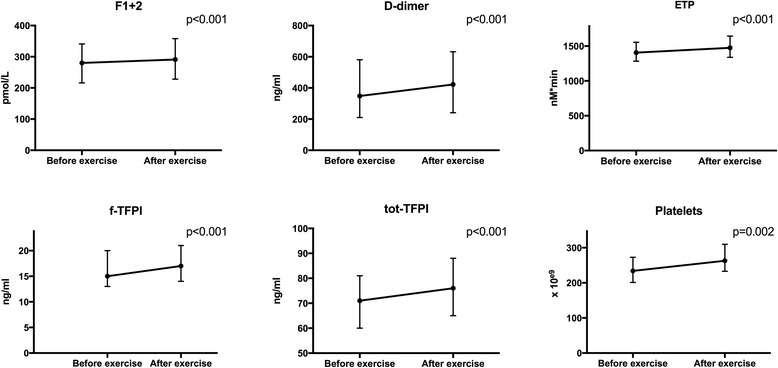
Levels of haemostatic markers before and after exercise in the total population (unadjusted for hematocrit). *P*-value refers to change from before to after exercise. The error bars on graphs represents 25th and 75th percentiles

Baseline levels of the haemostatic markers in the total population and according to having CAD or not are shown in Table 2. We observed a significant increase during exercise testing in most of the measured markers in the total population (*p* ≤ 0.002 for all) (Figure 1). When adjusting for change in hematocrit, the increase remained significant in all markers presented in Figure 1, except for D-dimer (*p* = 0.071). Median value of change in hematocrit during exercise was 0.03 (0.02, 0.04) units. As for the CAT assay parameters, only ETP was found to increase significantly (Fig. 1). LT, ttPeak and PeakH changed during exercise however, results were not significant after adjustment for hematocrit (data not shown). Velocity index did not change.

**Table 2:** Baseline haemostatic markers in the total population and stratified into groups according to angiographically verified CAD or not

	Overall (*n* = 106)	CAD (*n* = 70)	No CAD (*n* = 36)	*P*-value
Hb (g/dL)	14.6 (13.6, 15.1)	14.7 (13.6, 15.2)	14.3 (13.4, 15.1)	0.481
Hematocrit (units)	0.43 (0.40, 0.44)	0.43 (0.40, 0.44)	0.42 (0.40, 0.44)	0.711
Leucocytes (x 10^e9^)	6.60 (5.80, 7.90)	6.4 (5.8, 7.8)	6.8 (5.7, 8.3)	0.502
Platelets (x 10^e9^)	234 (201, 273)	222 (192, 261)	251 (232, 294)	*0.003*
Lactate (mmol/L)	1.40 (1.20, 2.00)	1.4 (1.2, 2.0)	1.4 (1.0, 2.0)	0.759
LT (min)	3.09 (2.68, 3.67)	3.0 (2.7, 3.3)	3.2 (2.9, 3.7)	0.301
ETP (nM*min)	1406 (1283, 1554)	1397 (1281, 1546)	1420 (1279, 1581)	0.667
PeakH (nM)	271.2 (243.3, 307.8)	265 (246, 308)	280 (233, 321)	0.662
ttPeak (min)	5.68 (5.17, 6.36)	289 (247, 329)	286 (253, 324)	0.346
Velo (nM/min^−1^)	103 (87, 132)	103 (88, 131)	110 (82, 135)	0.949
F1 + 2 (pmol/L)	280 (216, 341)	282 (216, 338)	275 (214, 359)	0.679
D-dimer (ng/ml)	348 (210, 581)	381 (221, 583)	296 (196, 578)	0.412
Free TFPI (ng/ml)	16 (13, 20)	15 (13, 19)	16 (12, 20)	0.741
Total TFPI (ng/ml)	71 (60, 81)	67 (58, 79)	77 (64, 84)	*0.027*

**Fig. 2:**
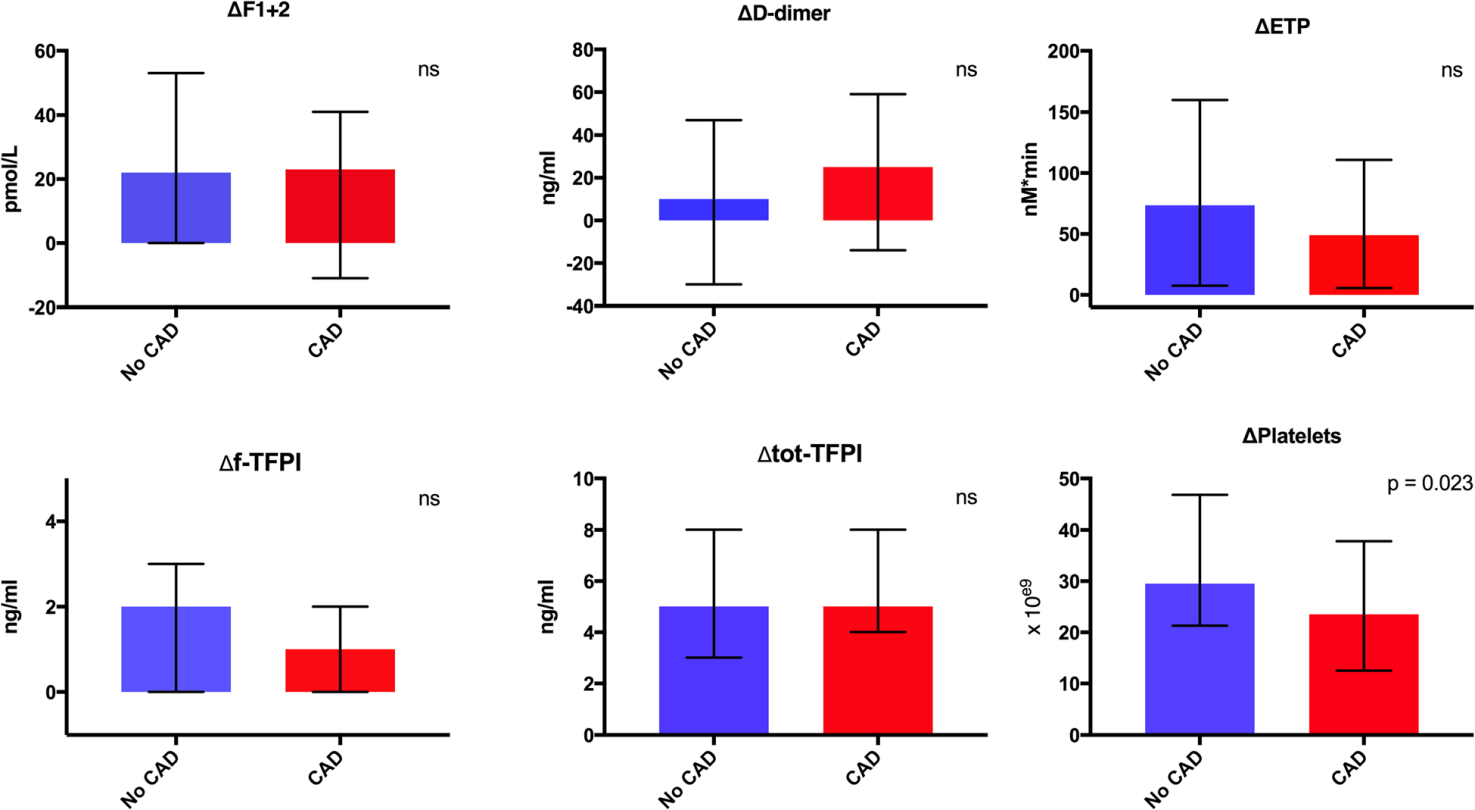
Changes in haemostatic markers during exercise in patients with angiographically verified CAD and no CAD (unadjusted for hematocrit). *P*-value refers to difference in change between groups. The error bars on graphs represents 25th and 75th percentiles. *ns* = non-significant

In patients with verified CAD compared to patients without CAD, significantly lower baseline levels of total TFPI (median value 67.4 versus 76.6 ng/ml respectively, *p* = 0.027) and platelet counts (median value 222 x 10^e9^ and 251 x 10^e9^ respectively, *p* = 0.003) were found (Table 2). Platelet counts were also significantly less increased during exercise in patients with CAD during exercise (*p* = 0.023), also when adjusted for hematocrit (*p* ≤ 0.001) (Figure 2). There were, however, no other significant differences in changes of the measured markers during exercise, between the two groups (Fig. 2). Neither was there any significant difference in changes of these markers comparing patients that were revascularized versus not (data not shown).

In patients with the longest time of exercise duration (≥12:46 min), according to the highest quartile, there was a significant increase in free TFPI during exercise. In this group, patients with CAD had significantly lesser increase in levels of free TFPI compared to patients without CAD (median value 0.4 and 1.6 ng/ml respectively, *p* = 0.047). However these results did not remain significant after adjustment for hematocrit (*p* = 0.09).

Otherwise, no other variables were related to exercise duration.

## Discussion

Our study demonstrates that among patients with symptoms of CAD there was a significantly increased pro-coagulant activity during short-term strenuous exercise. Our results show that free and total TFPI, ETP and F1 + 2 increased considerably during exercise and these results remain significant despite acknowledged hemoconcerntration during physical exercise. This considered hypercoagulable state was, however, not more pronounced in patients who were angiographically diagnosed with CAD or in need of revascularization compared to those without verified CAD.

It might be suggested that TFPI, being the main inhibitor of TF, would be consumed during a hypercoagulable state, such as in acute coronary syndrome (ACS) or strenuous exercise, or even to an increased extent when the two coincide. To our knowledge the present study is the first to report on the effect of exercise on TFPI in patients with CAD, and we have demonstrated a significant increase in both free and total TFPI. As TFPI is released from endothelial cells, under different stimuli, it has been claimed to be released upon endothelial activation [20]. Our results could therefore be discussed to be related to exercise induced endothelial activation.

However, some studies have shown lower levels of TFPI in patients with CAD, as also was found in our study [21–23]. Arguments of possibly wide inter-individual variation of TFPI levels as well as the known association of TFPI to cardiovascular risk factors or influence of use of medication may be responsible for the diverging data [15, 24]. Two previous studies have concluded that subjects with lower levels of TFPI have an increased risk of developing ACS over a 5-year period [25, 26]. This supports our findings of lower baseline TFPI levels in patients with CAD, considering that patients who developed ACS most likely have atherosclerotic arterial changes long before their first cardiovascular event. Other studies including patients with stable angina pectoris have shown that TFPI levels mostly do not differ from controls [15, 16, 27]. These varying data necessitate further research in this field.

Exercise intensity rather than exercise duration has been supported to affect the haemostatic profile [7, 9]. Our results on increased pro-thrombotic markers, F1 + 2 as well as increased ex vivo thrombin generation shown by ETP, are partly in line with previous studies. The borderline significant increased levels of D-dimer might also be discussed as part of the acute phase response. Several previous studies have established that healthy, well-trained individuals have lower ETP levels and higher F1 + 2 levels than untrained subjects [28–30]. This has partly been explained by a decrease of ex vivo thrombin generation in the presence of decreasing amounts of prothrombin by its conversion to thrombin in vivo, potentially an exhaustion phenomenon [31]. This did not correspond to findings in our CAD population as our results showed an increase in both markers, which might be discussed along with the exercise performance of relatively short duration. No data on exercise habits of our population was gathered; hence this might have influenced our results. Similar exercise performance was seen in both groups (CAD vs. no CAD), however this does probably not reflect upon exercise capacity of the individuals as they had different reasons for terminating the exercise test. Our finding of lower maximal heart rate in patients with CAD may be explained by worse chronotropic response due to CAD, premature limitation of exercise test due to ECG changes and symptoms or, most likely, by the significantly increased use of beta blockers in this group. Beta blocker use may also explain the overall significantly lower platelet counts in patients with CAD. Catecholamine activation leading to splanchnic activation and subsequent platelet release might be suppressed due to higher incidence of chronic use of beta blockers in the CAD patients [32, 33].

## Conclusion

Selected markers of pro- and anticoagulant activity investigated in our study on patients with symptoms of CAD, confirm a haemostatic activation with hypercoagulable response during strenuous exercise, however, this was not more pronounced in patients who presented with angiografically verified CAD.
